# A pilot placebo-controlled, double-blind, and randomized study on the cognition-enhancing benefits of a proprietary chicken meat ingredient in healthy subjects

**DOI:** 10.1186/1475-2891-12-121

**Published:** 2013-08-15

**Authors:** Zain M Azhar, Jamil O Zubaidah, Khin ON Norjan, Candy Yi-Jing Zhuang, Fai Tsang

**Affiliations:** 1 Department of Psychiatry, Faculty of Medicine & Health Sciences, Universiti Putra Malaysia, Serdang, Selangor 43400UPM, Malaysia; 2 BRAND’S® Health Science Centre, Cerebos Pacific Limited, 3 Biopolis Drive, #06-19, Synapse 138623, Singapore

**Keywords:** Essence of chicken, Chicken meat extract, Working memory, Attention, Cognition

## Abstract

**Background:**

It has long been postulated that the relative abundance of specific nutrients can affect cognitive processes and emotions. Newly described influences of dietary factors on neuronal function and synaptic plasticity have revealed some of the vital mechanisms that could be responsible for the action of diet on brain health and cognitive function. Here, through a double-blind, randomized, placebo-controlled trial, we asked if the newly discovered chicken meat ingredient-168 (CMI-168) could be beneficial to the cognitive function in healthy adults.

**Methods:**

Normal, healthy subjects were supplemented with either placebo or CMI-168 for 6 weeks. The subjects were given a series of cognitive tests to examine their levels of cognitive functioning at the beginning and end of supplementation, as well as two weeks after termination of supplementation. The combination of these tests, namely Digit Span Backwards, Letter-Number Sequencing, and the Rey Auditory Verbal Learning Test (RAVLT), was used to assess the subjects’ attention and working memory. For all comparisons, the probability level of *p* < 0.05 was taken as statistically significant using repeated measure 2-way ANOVA followed by Bonferroni post-hoc test.

**Results:**

Overall, subjects supplemented with CMI-168 showed significantly (*p* < 0.01) better performance in all cognitive tests after 6 weeks’ supplementation compared to control and such superior performance was maintained even 2 weeks after termination of supplementation.

**Conclusions:**

The present study reveals the cognition-enhancing properties of a recently developed chicken meat ingredient, likely arising from the promotion of attention and prefrontal cortex functions.

## Background

To prosper and flourish in a rapidly changing world, we must make the most of all our resources – both mental and physical. Added to highly competitive work lives are the rising demands from evolving family structures and increased social responsibilities [1]. Consequently, long working hours and disturbed work-life balance are common and these predispose individuals to long-term risk of lifestyle-related disorders [2,3]. Importantly, apart from the long-term health issues, productivity at work could also be impaired as a result of work stress and fatigue. As such, the use of cognitive enhancers is particularly appealing. The term "cognitive enhancement" usually characterizes interventions in humans that aim to improve mental functioning beyond what is necessary to sustain or restore good health. In spite of recent evidence supporting their efficacies in cognitive enhancement, the potential use of drugs to enhance cognition, emotion and executive function has raised debate on their ethical and safety concerns [1,4]. On the other hand, although food has classically been perceived as means to provide energy and building materials to the body, its ability to prevent and protect against diseases is starting to be recognized. In particular, over the past decade, research has provided exciting evidence for the influence of dietary factors on maintenance of cognitive function [5,6]. Dietary factors can affect multiple brain processes by regulation of neurotransmitter pathways, synaptic transmission, membrane integrity and signal transduction pathways [7,8]. However, despite a substantial body of pre-clinical evidence supporting the efficacies of foods and supplements in cognition enhancement, their efficacies in humans are yet to be conclusive.

Essence of Chicken (EOC) is an aqueous extract of chicken meat with a long history of consumption across Asian populations. Anecdotal evidence has long associated EOC with improving cognitive performance, especially related to learning and memory, as well as executive function. Published research over the last decade has lent significant support to its benefits in cognitive performance, particularly in the area of working memory, attention and episodic memory [9-11]. EOC, that is abundant in proteins, amino acids and peptides, is prepared through a unique process that involves aqueous extraction of chicken meat at a specific environment (e.g. temperature). Besides, it also contains bioactive peptides such as carnosine and anserine which are effective antioxidants and could have other physiological benefits to human health. This prompted further research to identify potential bioactives that could have cognition-enhancing benefits and resulted in the development of a hydrolyzed chicken extract, namely chicken meat ingredient-168 (CMI-168), designed for cognition-enhancing benefits. Here, we aimed to characterize the cognition-enhancing effects of CMI-168 in a group of healthy subjects. Subjects were supplemented with either placebo or CMI-168 for 6 weeks. Cognitive performance of the subjects was examined using a battery of cognitive tasks selectively assessing their working memory and attention immediately before and after the supplementation, as well as two weeks after the course of supplementation. This post-supplementation assessment was helpful to determine whether the benefits (if any) could be a result of sustainable optimization of cognitive function by CMI-168.

## Methods

### Subjects

A total of 46 healthy male and female subjects aged between 35 and 65 years were recruited either as walk-in or referred from their general practitioners for counseling for life-style related issues. They were not suffering from any medical condition and did not require any medication. Their psychological well-being was assessed using General Health Questionnaire, Beck Anxiety Inventory, Beck Depression Inventory, and Sheehan Disability Scale and confirmed that they did not suffer from other psychiatric disorders or serious medical illnesses. The study adhered to the strict standard Good Clinical Practice (GCP) of Malaysia and also Guidelines of Declaration of Helsinki in dealing with human subjects. Compliance of subjects was monitored by requesting them to bring the unfinished or empty bottles to exchange for new ones. Data from subjects non-compliant to the supplementation instructions was not included for statistical analyses. At the end of the study, a total of 26 subjects were excluded from the data analyses due to either non-compliance of supplementation or withdrawal from the study. The remaining fully compliant subjects consisted of 10 male and 10 female subjects respectively. Each supplementation group consisted of 5 male and 5 female subjects. Subjects were also required to provide a dietary diary throughout the study to determine if there were any substantial variations among the subjects, as well as any changes in the dietary profiles throughout the study. All the subjects were on mixed diets that included protein intake from sources such as eggs and meats from their normal diets.

### CMI-168

Chicken meat ingredient-168 (CMI-168) was a hydrolyzed chicken extract prepared from chicken meat that had been processed by a proprietary technology, involving bio-processing and aqueous extraction. It was produced through the advancement and optimization of the process used for production of essence of chicken (EOC).

### Test sample

Tablets containing the chicken meat ingredient-168 (CMI-168) (335 mg per tablet) were prepared. Subjects were supplemented with 2 tablets per day (i.e. 670 mg per day).

### Placebo

Tablets containing, instead of CMI-168, 335 mg of microcrystalline cellulose were prepared. Subjects were supplemented with 2 tablets per day as the CMI-168-supplemented group.

Both test sample and placebo were supplied by Cerebos Pacific Limited, Singapore.

### Study design

A randomized, double-blind, placebo-controlled study was conducted to evaluate the effect of CMI-168 on cognitive performance. Subjects were randomly divided into 2 groups, namely the placebo group and CMI-168 group. Subjects were supplemented orally with either the placebo or test sample daily for 6 weeks. The subjects and the investigator who conducted the tests were blinded to the information about the group allocation and samples provided. Instead, an independent investigator, who has no information about the cognitive assessment and psychological well-being, maintained the record of all the samples and group allocation. During the 6 weeks of supplementation that subjects self-administered the supplements (either placebo or CMI-168), the subjects were required to return the unfinished supplements or empty bottle before they were issued with new bottles of supplements. There were about 20 subjects who did not fully comply with the supplementation regime and their data were excluded from the final statistical analyses.

### Measurements

The subjects were examined using a battery of psychological assessments as well as cognitive performance tasks at the beginning of study (day 0) and day 42 (week 6). They were also tested on day 56 (week 8) to continue monitoring the effects of CMI-168 on cognitive performance after termination of supplementation. This post-supplementation assessment of cognition was intended to provide information on the sustainability of the cognition-enhancing benefits of CMI-168. The psychological assessments were Beck Anxiety Inventory, Beck Depression Inventory, General Health Questionnaire, Sheehan Disability Scale; the cognitive performance tasks were Digit Span, Letter Number Sequencing and Rey Auditory Verbal Learning Test (RAVLT). These pre- and post-supplementation psychological assessments were helpful to ensure that the subjects are free from any psychological or psychiatric disorders at beginning of the study and there was no negative psychological effect on the subjects as a result of the supplementation.

### Beck Anxiety Inventory and Beck Depression Inventory

Beck Anxiety Inventory (BAI) is a 21 item self report scale designed to measure the severity of anxious symptoms [12]. The score ranges from 0–63. The test-retest reliability is 0.75 and concurrent validity is 0.65 with Hamilton rating score. Beck Depression Inventory (BDI) is another self report scale that measures the severity of depression symptoms [13]. The score ranges from 0–63. Any score above 30 would indicate severe depression. The normal range is 0–9. The test-retest reliability is 0.8 and it has a high validity index.

### General Health Questionnaire

General Health Questionnaire (GHQ) is a structured questionnaire validated for use in Malaysia in 1996 [14]. It has become a commonly used instrument in multicentre, international trials designed to detect psychiatric disorders, particularly, states of depression, anxiety and psychiatric morbidity.

### Sheehan Disability Scale

The Sheehan Disability Scale (SDS) was developed to assess functional impairment in three inter-related domains; work/school, social and family life [15]. It is a brief self-report tool with a 10 point visual analogue scale. Scores above 5 are associated with significant functional impairment.

### Digit Span Backwards

The Digit Span assessment typically consists of Digit Span Forwards and Digit Span Backwards. The Digit Span Forwards is generally simpler task and is a measure of short-term memory storage capacity. In contrast, as the Digit Span Backwards [16] requires the subject to recall and repeat auditory information in the reverse sequence, it not only requires the short-term memory storage capacity, but also involves the manipulation of information within the phonological buffer. As such it is also a measure of working memory. As one of our key objectives of the current study is to understand how CMI-168 could be beneficial to the cognitive resource optimization for effective information processing and manipulation, Digit Span Backwards task was employed in this study to more selectively examine the working memory. The subjects were presented with a series of digits and were asked to immediately repeat them verbally in reverse order. The length of the longest list a subject can remember is that subject’s digit span.

### Letter-Number Sequencing

Letter-Number Sequencing (LNS) requires the subject to attend to a series of letters and numbers that have been read to him or her, hold them in memory, manipulate them into a new order, and repeat the sequence. It was indicated while much of the variance on the LNS task was explained by performance on the more traditional Digit Span, additional unique prediction of LNS performance are provided by measures of processing speed and visual spatial working memory [17].

### Rey Auditory Verbal Learning Test

The Rey Auditory Verbal Learning Test (RAVLT) is an efficient neuropsychological instrument for evaluating verbal memory and learning. It provides scores for assessing immediate memory, new verbal learning, susceptibility to interference, retention of information after a period of time, and memory recognition [18,19]. Briefly, it consists of 3 lists. The first list consists of 15 words and participants are allowed 3 attempts to remember as many words as possible (termed ‘Immediate Memory’ , ‘Best Learning’ and ‘Total Learning’ respectively). Participants are then presented with a distractor list (List B) followed by a free-recall test from List B (termed ‘Proactive interference’). Immediately after, participants are asked to recall List A (termed ‘Retroactive interference’). After a 20 min interval, participants are asked again to recall the words from List A (termed ‘Delayed Recall’). Finally, the last task presents a 3^rd^ list (List C) which includes words from List A. The participants need to identify the words that are part of List A (termed ‘Recall’).

### Statistics

Requirement of the sample size to provide statistical significance was computed using Epi Info™ 7 software with the anticipated effect size was 0.8, power of 0.8 and confidence level of 95%. Statistical Package for Graphpad Prism was used to analyze the data collected in this study. All values are expressed as Score for each test. All values were expressed as mean ± standard error of mean (SEM). For all comparisons, the probability level of *p* < 0.05 is considered to be statistically significant using 2-way repeated measures ANOVA followed by Bonferroni post-hoc test.

## Results

### Sample characteristics

Two groups of subjects consisting of a total of 46 subjects were recruited for this study. The final number of subjects whom the data was examined for statistical differences was 20. The reason for exclusion from statistical analyses were either withdrawal from the study or non-compliant to the supplementation regime. The sample size was verified using Epi Info™ 7 software to ensure the sample size is sufficient for detecting differences between placebo and CMI-168 supplementations. Among the 20 subjects, their ages ranged from 35 to 65 years old (Mean age: 47.5 years old). Each group consisted of 5 male and 5 females. None of the participants reported any adverse effects during and after the course of supplementation. For concealed allocation purpose, the investigator ruling participants eligible for the trial did not know their group allocation when determining eligibility.

### Psychological profiles

There were no significant differences between the two groups in the Beck Anxiety Inventory and Beck Depression Inventory (data not shown). These psychological assessments confirmed that the subjects were normal and free of any psychiatric disorders. From the clinical assessment by the qualified psychiatrist in charge of this clinical study, the subjects were only mildly stressed due to their social or physical environments. Throughout the study, there was also no significant difference between the 2 groups in General Health Questionnaire and Sheehan Disability Scale, which also confirmed that their psychological status was unchanged during the study.

### Cognitive assessments

#### (1) Attention & working memory

**Figure 1:**
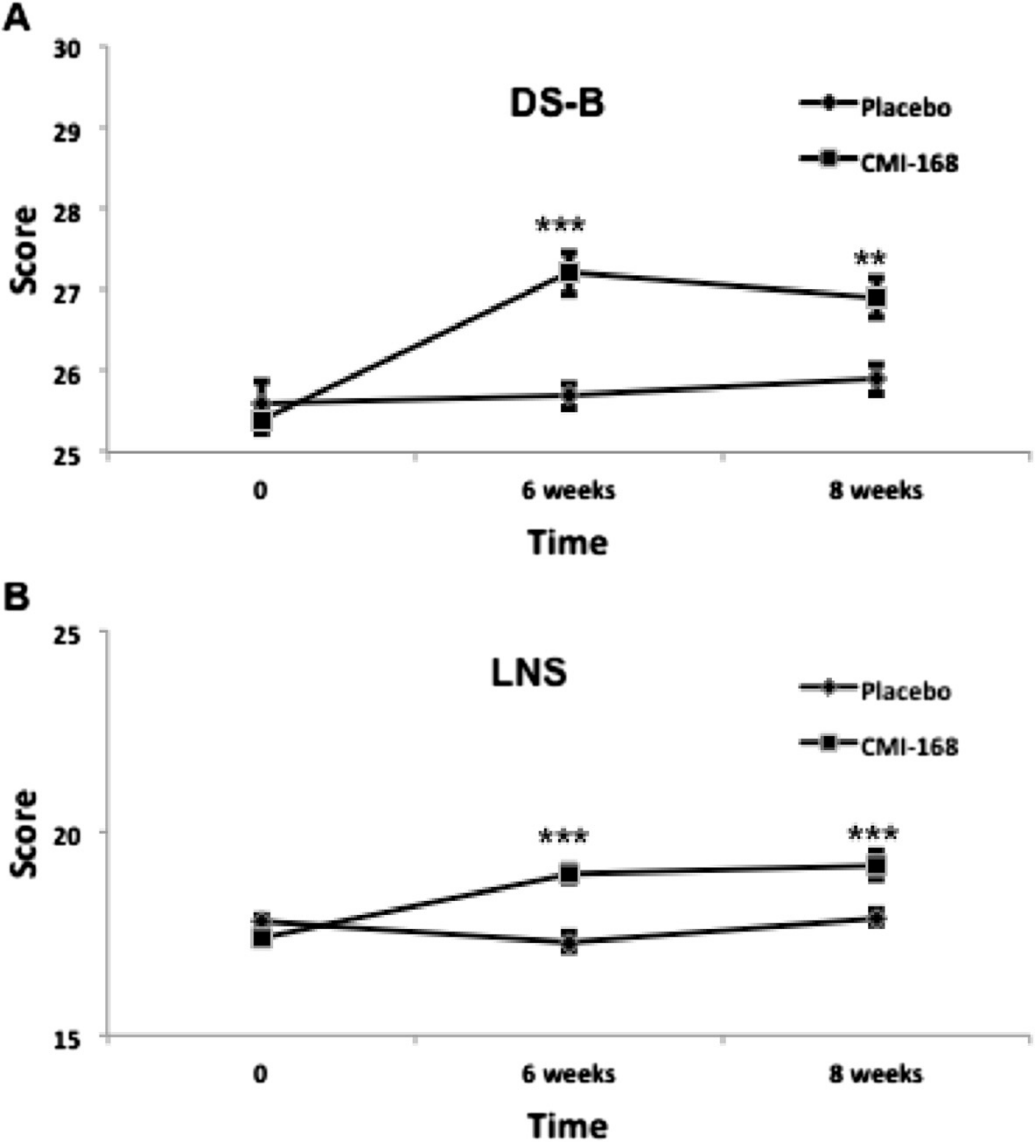
**Effects of a proprietary chicken meat ingredient-168 (CMI-168) on working memory as examined by (A) Digit Span Backwards, DS-B; and (B) Letter-Number Sequencing, LNS.** Each data point is represented as mean ± S.E.M. (n = 10). ** *p* < 0.01, *** *p* < 0.001, compared with placebo group at the same time point.

The Digit Span consists of two parts, Digits Forward and Digits Backwards. It is also known as auditory vocal sequencing memory that requires the subjects to recall and repeat auditory information in the proper sequences. The performance scores of Digits Forward test were recorded as a clinical note and throughout the study there was no significant change (*p* > 0.05; data not shown) in all subjects in their basic short-term memory storage capacity. Both Digit Span Backwards and Letter-Number Sequencing are useful in examining the subjects’ attention and working memory. As shown in Figure 1, after a supplementation of 6 weeks, subjects taking CMI-168 showed a higher score compared to those taking placebo in both the Digit Span Backwards and Letter Number Sequencing tasks (*p* < 0.001). The better performance in CMI-168 group was maintained even 2 weeks (week 8) after termination of supplementation (*p* < 0.01).

#### (2) Verbal memory and learning

**Figure 2:**
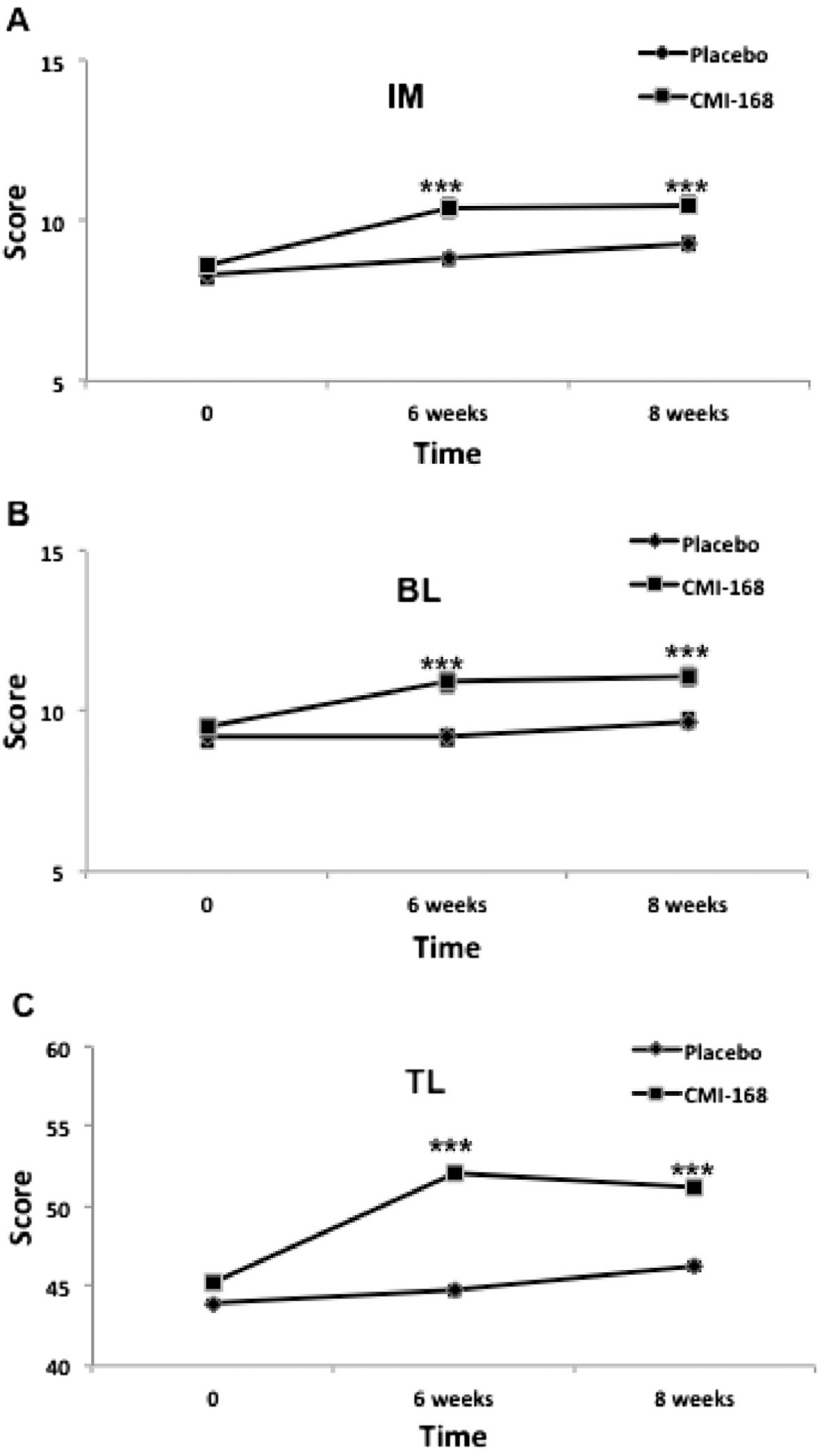
**Effects of a proprietary chicken meat ingredient-168 (CMI-168) on verbal memory and learning as examined by RAVLT.** New verbal learning scores were reflected by **(A)** Immediate Learning, IL; **(B)** Best Learning, BL; and **(C)** Total Learning, TL. Each data point is represented as mean ± S.E.M. (n = 10). *** *p* < 0.001, compared with placebo group at the same time point.

**Figure 3:**
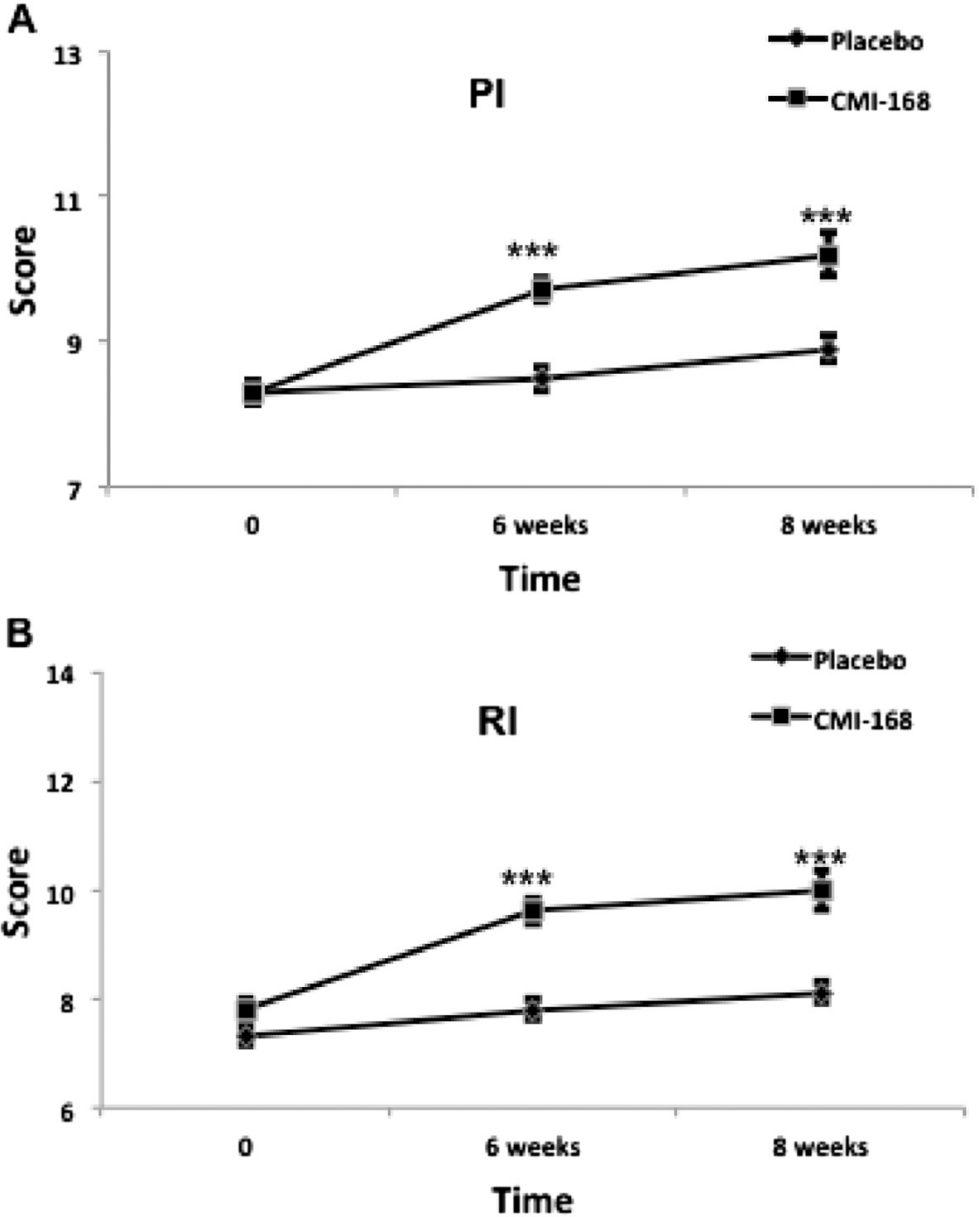
**Effects of a proprietary chicken meat ingredient-168 (CMI-168) on verbal memory and learning as examined by RAVLT.** Susceptibility to interference was determined by **(A)** Proactive Interference, PI; and **(B)** Retroactive Interference, RI. Please refer to Methods for detailed description of the assessments. Each data point is represented as mean ± S.E.M. (n = 10). *** *p* < 0.001, compared with placebo group at the same time point.

**Figure 4:**
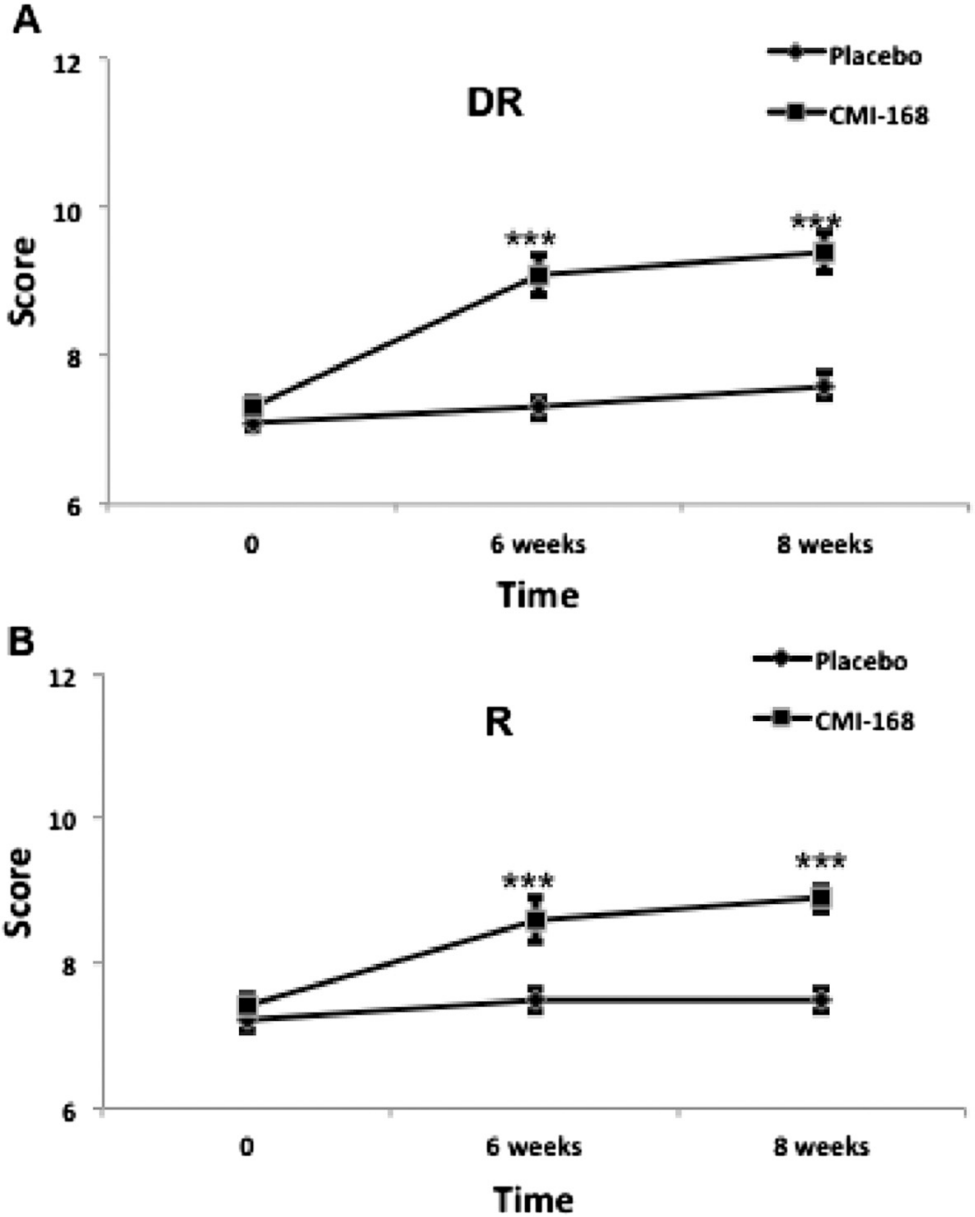
**Effects of a proprietary chicken meat ingredient (CMI) on verbal memory and learning as examined by RAVLT.** Memory retention and recognition were determined by **(A)** Delayed Recall, DR; and **(B)** Recall, R respectively. Each data point is represented as mean ± S.E.M. (n = 10). *** *p* < 0.001, compared with placebo group at the same time point.

The Rey Auditory Verbal Learning Test (RAVLT) was used to assess verbal memory and learning. Particularly, it is useful for assessments of immediate memory related to new verbal learning and the capability to retain information for effective recall. The scores of verbal learning and memory on the RAVLT also correlate strongly with executive function. Figure 2 shows that subjects taking CMI-168 scored significantly higher (*p* < 0.001) compared to placebo group in the RAVLT subtests of Immediate Memory, Best Learning and Total Learning. In the assessment of the susceptibility to interference, subjects supplemented with CMI also demonstrated more robust (*p* < 0.001) memory despite interference (proactive and retroactive) compared to control (Figure 3). Further, the efficiency of information retention (as assessed by Delayed Recall) was shown to be higher (*p* < 0.001) in subjects supplemented with CMI-168 compared with control (Figure 4A). Consistently, the memory recognition, which is also a function of memory retention, the CMI-168-supplemented group was also significantly (*p* < 0.001) superior relative to the control group (Figure 4B).

Taken together, subjects supplemented with CMI-168 for 6 weeks showed better overall performance scores in all cognitive tests and their superior performance was maintained even 2 weeks after termination of supplementation. In the current study, the differences in cognitive functions between male and female were not determined as this was intended as a pilot study on the cognition enhancing effects of CMI-168 and the sample size was not large enough to provide a conclusive comparison between genders.

## Discussion

In this study we explored the cognition-enhancing effect of a proprietary chicken meat ingredient, CMI-168. Previous studies on EOC have demonstrated its benefits in the maintenance of executive function under stress [9-11], particularly in attention and working memory. From an initial study intending to explore the mechanism(s) through which EOC might work in the brain to regulate cognitive function, we found that EOC seemed to have modulatory effects in the serotonergic system in the brain [20]. This prompted us to further explore into the bioactive ingredients that are responsible for the observed benefits in cognitive function. Consequently, in an attempt to engineer a more efficacious ingredient through optimization of the proprietary process that produces EOC, a chicken meat ingredient, CMI-168, was developed. Interestingly, preliminary in vivo assessment of CMI-168 indicated that it could promote more selectively the learning and memory functions of the mice [data not shown]. Naturally, one of the key questions would be to ask if this selective efficacy of enhanced learning and memory could be reproduced in humans. This data, together with further in-depth mechanistic studies in vitro and in vivo, will then provide a more encompassing understanding of the cognition-enhancing properties of CMI-168.

As shown in Figure 1, subjects supplemented with CMI-168 for 6 weeks showed significantly (*p* < 0.001) better scores in both Digit Span Backwards and Letter-Number Sequencing tests. A better performance in these tests suggests a better ability in sequencing as well as a higher level of attention and concentration. Besides, as both tests require manipulation of information within the phonological buffer, they are measures of working memory as well. This finding is in line with previous reports by Azhar and colleagues [8,9] and indicates that the cognition-enhancing effect of CMI-168 correlates well with that of EOC. We further examined, using RAVLT, the subjects’ verbal learning and memory capabilities. In the immediate recall tests, subjects supplemented with CMI-168 showed significantly better performance (Figure 2). This could result from an enhanced ability to concentrate. This possibility is further supported by the results of the subsequent sub-test on the memory despite interference where the CMI-168-supplemented group again performed significantly better than the placebo group (Figure 3). Furthermore, previous studies using RAVLT also suggest that interferences (i.e. proactive and retroactive interferences) could have impact on both the normal functioning of working memory and memory processes such as consolidation and storage [21-26]. Therefore, this suggests efficacy for CMI-168 in the maintenance of the proper functioning of working memory as well as important memory functions such as memory consolidation. Importantly, these processes are pivotal contributory factors to effective learning. Further supporting the notion that CMI-168 would be particularly beneficial to learning, in the last part of RAVLT that assess the efficiency of information retention through the delayed recall and memory recognition tasks (Figure 4), CMI-supplemented group showed a consistently superior performance compared to placebo group. To help rule out the possibility of a temporary equilibrating adjustment of function due to supplementation of CMI-168, rather than a sustainable improvement of cognitive function, we conducted another cognitive assessment two weeks after termination of supplementation. Interestingly, in the post-supplementation cognitive assessment, the CMI-168-supplemented group maintained higher scores of performance in all tests in comparison with the placebo group. Understanding that the central activities of CMI-168 are associated with enhancement of effective neuronal communications based on the in vitro and in vivo findings (data not shown), it is possible that supplementation of CMI-168 might help optimize the cognitive resources for effective PFC processing and attentional network. This optimization of PFC function would provide an advantage in the learning process, particularly related to information acquisition, processing and manipulation that are essential for conceptualization and application of newly registered information. Additionally, the ability to maintain effective memory functions such as memory consolidation would be an important determinant of successful storage of newly learned information and subsequent retrieval for application in future. Thus it is plausible to hypothesize that CMI-168 helps to maintain an effective network of cognitive resources, particularly beneficial in the maintenance of attention, working memory, as well as memory functions such as memory storage and consolidation. This hypothesis warrants further investigation in the application of CMI-168 on the strengthening of the neural networks and functional connectivity related to PFC and hippocampal functions. Although the present study was not intended to investigate any differential responsiveness to CMI-168 in the subjects, it does not rule out the possibility that CMI-168 might have variation in the magnitude of impact across individuals.

## Conclusion

The present study reveals initial evidence supporting the cognition-enhancing efficacy of a proprietary chicken meat ingredient, CMI-168, in humans. This benefit might be associated with its ability to maintain effective cognitive resources in attention- and PFC-related executive functions, as well as memory processes that facilitate consolidation and storage of newly learned information. This initial evidence warrants further investigation into the selectivity of the effects of CMI-168 in specific cognitive processes in the prefrontal cortex, as well as hippocampus in the learning process.

## Competing interest

CYJZ and FT are employees of Cerebos Pacific Limited, Singapore.

## Authors’ contributions

ZMA designed and supervised the study; JOZ and KONN conducted the study and performed analyses of the data; CYJZ coordinated the preparation and delivery of the test materials to the test site; FT prepared the manuscript; ZMA reviewed the manuscript; all authors read and approved the final manuscript.
